# CD8 T cells are dispensable for experimental autoimmune prostatitis induction and chronic pelvic pain development

**DOI:** 10.3389/fimmu.2024.1387142

**Published:** 2024-05-14

**Authors:** Florencia C. Salazar, Maria S. Martinez, Daniela A. Paira, Yair A. Chocobar, Carolina Olivera, Gloria J. Godoy, Eva V. Acosta-Rodriguez, Virginia E. Rivero, Ruben D. Motrich

**Affiliations:** ^1^Centro de Investigaciones en Bioquimica Clinica e Inmunologia (CIBICI)-CONICET, Departamento de Bioquimica Clinica, Facultad de Ciencias Quimicas, Universidad Nacional de Cordoba, Cordoba, Argentina; ^2^Federation of Clinical Immunology Societies (FOCIS) Center of Excellence Centro de Inmunologia Clinica de Cordoba (CICC), Cordoba, Argentina

**Keywords:** autoimmunity, prostatitis, inflammation, CD8 T cells, chronic pelvic pain, animal model, pathogenesis

## Abstract

**Introduction:**

Chronic Pelvic Pain Syndrome or Chronic Prostatitis (CPPS/CP) is the most prevalent urologic affliction among young adult men. It is a challenging condition to treat, which significantly decreases patient quality of life, mostly because of its still uncertain aetiology. In that regard, an autoimmune origin is a prominent supported theory. Indeed, studies in patients and in rodent models of Experimental Autoimmune Prostatitis (EAP) have provided compelling evidence suggesting a key role of CD4 Th1 cells in disease pathogenesis. However, the implication of other prominent effectors of the immune system, such as CD8 T cells, has yet to be studied.

**Methods:**

We herein analyzed the induction of prostatitis and the development of chronic pelvic pain in EAP using CD8 T cell-deficient animals.

**Results:**

We found similarly elevated PA-specific immune responses, with high frequencies of specific IFNg+CD4+ and IL17+CD4+ T cells in prostate draining lymph nodes from PA-immunized either CD8 KO or wild type animals with respect to controls. Moreover, these peripheral immune responses were paralleled by the development of significant chronic pelvic pain, and accompanied by prostate histological lesions, characterized by hemorrhage, epithelial cell desquamation, marked periglandular leukocyte infiltration, and increased collagen deposition in both, PA-immunized CD8 KO and wild type animals. As expected, control animals did not develop prostate histological lesions.

**Discussion:**

Our results indicate that CD8 T cells do not play a major role in EAP pathogenesis and chronic pelvic pain development. Moreover, our results corroborate the previous notion that a CD4 Th1 associated immune response drives the induction of prostate tissue inflammation and the development of chronic pelvic pain.

## 1 Introduction

Chronic pelvic pain syndrome or chronic prostatitis (CPPS/CP) stands as the most prevalent urologic condition among males under 50 years of age and the third most common in older males, accounting for up to 25% of all outpatient visits and urology consults (1–3). It has a considerable negative impact on quality of life of patients, comparable to other major long-term health conditions such as Crohn´s disease, diabetes mellitus, angina, or myocardial infarction (4, 5). CPPS/CP manifests with a wide array of pain and inflammatory symptoms, varying in type and intensity, affecting regions such as the pelvis, rectum, perineum, penis, testes, and lower back, persisting for at least 3 of the preceding 6 months. Although in most patients pain is accompanied by semen and/or prostate inflammation, no invading infectious agents are detected (5–8). Since its aetiology and pathogenesis remain unclear, most therapies are empiric and ineffective (9, 10). In fact, the therapeutic options currently available for patients are inadequate and often fail to meet the expectations of both physicians and patients alike (9). In consequence, CPPS/CP is usually misdiagnosed and poorly managed, resulting in chronic signs and symptoms, substantial morbidity, and overall patient dissatisfaction despite being common in clinical practice (11). CPPS/CP seems to involve similar clinical phenotypes resultant from several heterogeneous conditions, and dysregulated inflammation as a consequence of autoimmunity against the prostate has been proposed to be involved in its onset and/or progression (1, 10, 12–14). Cumulative data obtained from the study of both, the human disease and rodent models of Experimental Autoimmune Prostatitis (EAP), have provided compelling evidence regarding the immune mechanisms that underlie the induction and development of the disease and its associated consequences (10, 15–17). Indeed, self-reactive T cell and antibody responses specific to prostate antigens (PA) in CPPS/CP patients have been reported (18–22). Interestingly, increased IFNγ-secreting Th1 cell responses specific to PA, concomitant with urogenital inflammation as well as reduced semen quality, have been detected in a substantial fraction of men suffering from CPPS/CP, thus indicating that PA-specific Th1 cell-mediated immune responses may contribute to the development of male reproductive tract inflammation and chronic pelvic pain (22–24). Besides, EAP animal models serve as valuable tools in investigating the pathogenesis of CPPS/CP, as they closely mimic key features of the human disease, demonstrating their reliability and utility (10, 12, 17). They have shown that, upon PA immunization, animals develop specific Th1 cell responses that are accompanied by important lesions and infiltration of the prostate (17, 25, 26). Cumulative evidence indicated a key role of CD4 Th1 cells in the induction of inflammation in the prostate gland and the development of chronic pelvic pain, the characteristic symptom observed in patients with CPPS/CP (27–32). Although they have provided important data on the pathogenesis of the disease, useful for the development of new and more rational therapies, the possible pathogenic role of other prominent effectors of the immune system like CD8 T cells has not been studied yet.

CD8 T lymphocytes are crucial for the elimination of tumor cells and intracellular pathogens, demonstrating functional plasticity and complexity (33, 34). However, it is currently known that CD8 T cells play a plethora of functions, encompassing direct and indirect cytotoxic and non-cytotoxic actions (35). Upon activation in secondary lymphoid organs following antigen presentation, naïve CD8 T cells undergo rapid clonal expansion, resulting in large numbers of antigen-specific acute effector and memory CD8 T cells. This process is intricately regulated by various cell types, including CD4 T cells, which contribute to establishing optimal niches for the development and maintenance of effective CD8 T cell immunity (36). Effector CD8 T cells migrate to primary sites of infection or tumors, where they secrete cytokines such as IFNγ and effector molecules like perforin and granzyme, aimed at specifically eliminating the target cells *in situ* (37). Most of these acute effector CD8 T cells are eventually eliminated following antigen clearance, leading to the formation of a residual pool of diverse memory cells (38). Memory CD8 T lymphocyte subsets are thought to be structured in a developmental hierarchy, where tissue stem cell memory CD8 T cells (T_SCM_) self-renew and give rise to long-lasting central memory T cells (T_CM_), effector memory T cells (T_EM_), and tissue resident T cells (T_RM_) (39). Most CD8 T cells found in peripheral tissues are genuine T_RM_, which establish particular niches in nearly every organ (40). Regarding that, in is interesting to highlight that CD8 T cells are commonly found infiltrating inflamed tissues in several autoimmune diseases (35, 38). Interestingly, increased levels of CD8 T cells have been reported in semen from CPPS/CP patients (24). Moreover, several researchers have reported significant CD8 T cell infiltration in the prostate from animals with EAP (29, 31, 41, 42), suggesting they could play a major role in disease induction and progression, and the consequent pelvic pain. However, to the best of our knowledge, there is currently no documented evidence regarding the assessment of these cells involvement in the pathogenesis of chronic prostatitis. Considering that, with the aim of expanding the current knowledge of the pathogenesis of CPPS/CP, we herein analyzed the role of CD8 T cells in the induction of prostate inflammation and the development of chronic pelvic pain using an animal model of EAP.

## 2 Materials and methods

### 2.1 Ethics statement

Animal studies were conducted under protocols EXP-UNC: 0002734/2016 and EXP-UNC: 60699/2018 approved by the Institutional Laboratory Animal Care and Use Committee (CICUAL) at Facultad de Ciencias Químicas, Universidad Nacional de Córdoba (FCQ-UNC). CICUAL conducts its reviews strictly adhering to the guidelines of the Canadian Council on Animal Care (CCAC), the Directive of the European Union on the protection of animals used for scientific purposes (2010/63/EU), and the recommendations outlined in the Guide for the Care and Use of Laboratory Animals by the NIH (NIH publication 86–23).

### 2.2 Mice and antigens

Male CD8α knock-out (B6.129S2-Cd8atm1Mak/J) (43) and C57BL/6 wild type mice were procured from Jackson Laboratory (Bar Harbor, ME, USA). The mice were kept and cared for under specific pathogen-free (SPF) conditions at the Animal Care Facility of the Facultad de Ciencias Químicas, Universidad Nacional de Córdoba, and were utilized at the age of 6–8 weeks. The animal care facilities and programs of CIBICI meet the requirements of the national laws and institutional guidelines that are based on NIH regulations. Our facility has been certified by OLAW-NIH, Animal Welfare Assurance Number F16–00193.

The preparation of PA and the purification of Prostatein, also known as Prostate Steroid-Binding Protein (PSBP), the main target autoantigen in EAP, were conducted following established protocols as previously described (29). Briefly, PA extracts were prepared from Wistar rat prostate glands. Pooled glands were homogenized in protease inhibitors-supplemented PBS in an Ultra-Turrax homogenizer. The homogenate was centrifuged at 100,000×*g* for 30 min, and the supernatant was used as PA mixture. Protein concentration was determined using the Folin phenol reagent. Aliquots were kept frozen at -20°C until use. PSBP was purified following the procedure described by Chen et al. (44). Ventral prostates were homogenized in 20 mM Tris-HCl using an Ultra-Turrax homogenizer, and the cytosolic fraction was obtained after 60 min of centrifugation at 100,000×*g* and 4°C. The resultant supernatant was applied onto a Mono-Q FPLC column. Proteins were eluted with a linear 0–60% NaCl gradient in 20 mM Tris-HCl. Protein concentration of each fraction was evaluated by measuring its OD280, which were run under non-denaturing conditions in 15% polyacrylamide gels (SDS-PAGE). Fractions containing two bands of 18 and 20 kDa (corresponding to both subunits of PSBP) were also run under denaturing conditions to verify their identity. The purity of the PSBP preparation was >95% evaluated by Western blot using a mouse mAb recognizing the C3 polypeptide (kindly given by Dr. P. Bjork, Pharmacia and Upjohn, Uppsala, Sweden); and was LPS free tested by Gel clot 0.03 endotoxin units/ml sensitivity (Charles River, Laboratories International, Wilmington, NY, USA).

### 2.3 Antibodies

The antibodies utilized in various experiments, along with their respective manufacturers, were as follows: anti-CD3 (145–2C11), anti-CD4 (RM4–5), anti-Gr1 (RB6–8C5), anti-CD11b (M1/70), anti- anti-IL-10 (JES5–16E3), IFNγ (XMG1.2), and IgG1 isotype controls were procured from BD Biosciences (San Diego, CA, USA). The following antibodies were obtained from eBioscience (San Diego, CA, USA): anti-CD45 (30-F11) and anti-IL-17A (eBIO17B7). Antibodies including anti-CD45 (30-F11), anti-CD19 (6D5), anti-CD11b (M1/70), anti-NK-1.1 (S17016D), anti-CXCR3 (CXCR3–173), and anti-CCR5 (HM-CCR5) were purchased from BioLegend (San Diego, CA, USA). The antibodies were conjugated with APC, FITC, PE, PerCP-Cy5.5, Alexa Fluor 647, or PE-Cy7 and appropriately paired.

### 2.4 EAP induction and histopathological score

Male CD8-KO and C57BL/6 wild-type mice, aged six to eight weeks, were subcutaneously immunized in the hind footpad and at the base of the tail. The immunization comprised PA (300 μg/mouse, PA-CD8 KO and PA-WT groups, respectively) or saline solution (control group, C-WT) emulsified in complete Freund’s adjuvant (CFA, Sigma-Aldrich, St. Louis, MO, USA) at a total volume of 150 μl/mouse, following established protocols (25, 26, 29). Mice were immunized on days 0 and 15 and subsequently euthanized on day 24 according to the experimental timeline. A total of 58 mice were analyzed; n=4–6 mice per experimental group, being experiments repeated at least thrice. EAP severity was evaluated through histological scoring, conducted in a double-blind manner as described previously (27, 29). The inflammation level was graded on a scale of 0–3: 0 for no inflammation, 1 for mild perivascular cuffing with mononuclear cells, 2 for moderate perivascular cuffing with mononuclear cells, and 3 for marked perivascular cuffing, hemorrhage, and abundant mononuclear cells in the parenchyma. Evaluation was performed on 5 μm thick prostate tissue sections from each organ per animal, processed using conventional hematoxylin and eosin staining. Slides were examined under a Nikon TE 2000U microscope (Nikon, Osaka, Japan), and images were analyzed using Adobe Photoshop CS6 version 13 image analysis software (Adobe Systems Inc., San Jose, CA, USA).

### 2.5 Collagen quantification by picrosirius red staining

Prostate tissue samples were fixed in 4% paraformaldehyde, dehydrated in alcohol, cleared in xylene, and infiltrated with paraffin. Subsequently, 5 μm sections were stained with PSR according to a previously established protocol (45). Fluorescent imaging was conducted utilizing a BZ-X710 digital microscope (Keyence, Itasca, IL) equipped with a 20X dry objective (PlanFluor, NA 0.45) and illuminated by full spectrum light filtered with Texas red and FICZ filters. Tiled images were captured using Keyence image acquisition software (Keyence, Itasca, IL) and stitched to create a comprehensive image of the entire prostate section. Specific collagen staining was isolated by subtracting tissue autofluorescence from the acquired images. The total tissue area was determined by outlining the perimeter of the prostate lobe using a freehand selection tool and measuring the pixel area of the selected region. Collagen density was assessed by quantifying the area of PSR staining relative to the total tissue area using CT-FIRE software (LOCI, Madison, WI).

### 2.6 Cell culture

Mononuclear cell suspensions were individually prepared from prostate draining lymph nodes of mice in HBSS (Sigma-Aldrich) using Ficoll-Paque PREMIUM 1.084 (GE Healthcare Bio-Sciences AB, Uppsala, Sweden) centrifugation gradients. Live cells were counted via Trypan blue exclusion, then resuspended in RPMI 1640-GlutaMAX medium (Life Technologies, Carlsbad, CA, USA) supplemented with 1% penicillin/streptomycin (Life Technologies), 50 mM 2-ME (Life Technologies), and 10% FCS (Life Technologies). They were cultured in round-bottomed plates either with PSBP (20 μg/ml) or medium alone. Plates were incubated at 37°C in a water-saturated 5% CO2 atmosphere. Subsequently, cells were subjected to surface and/or intracellular cytokine staining and analyzed using FACS.

For lymphoproliferation assays, cells were seeded at a density of 3x10^5^ cells per well in a volume of 0.2 ml in flat-bottomed 96-well plates. Cell were cultured in the presence of PSBP (20 μg/ml) or medium alone. All cell combinations were replicated in quadruplicate. Plates were then incubated for 4 days at 37°C in an atmosphere saturated with 7.5% CO2 and pulsed during the final 18 hours with 1 μCi [methyl-3H] thymidine per well. The labelled material was automatically harvested and counted using a β-plate scanner. The response was quantified as a proliferation index, computed from counts per minute (cpm) incorporated in antigen-pulsed cultures divided by cpm incorporated in cultures with medium alone, as described previously (29).

### 2.7 Quantification of PSBP-specific antibodies in serum

PSBP-specific serum levels of total IgG, IgG1, and IgG2a were evaluated using conventional ELISA methods as detailed previously (29). In detail, multiwell plates (Maxisorp) were coated with 50μl/well of PSBP (20 μg/ml) in 0.05 M carbonate buffer pH 9.6 and left to incubate overnight at 4°C. Following this, microwells were washed twice and subsequently blocked with 3% BSA (Sigma-Aldrich) in PBS for 2 hours at 37°C. After rinsing with PBS-Tween 20 at 0.05%, 100 μl of serum (obtained post-cardiac puncture) serial dilutions (starting at 1/50) were added to the wells and allowed to incubate for 1 hour at 37°C. To identify specific total IgG, IgG1, and IgG2a, the plates underwent another washing step and were then incubated with a solution containing HRP-conjugated rat anti-mouse IgG, anti-mouse IgG1, or anti-mouse IgG2a (BD Biosciences) for 1 hour at 37°C. Following thorough washing, the reaction was developed with BD OptEIA TMB Substrate Reagent Set (BD Biosciences). Serum reactivity was quantified as OD measured at 450 nm using a microplate reader (Bio-Rad Laboratories, Hercules, CA, USA).

### 2.8 Flow cytometry

Prostate draining lymph node cells were stimulated with PSBP *in vitro*, followed by staining for cell surface markers and intracellular cytokines, according to established protocols (27, 29). In brief, cells were stimulated *in vitro* with PSBP (20 μg/ml) for 48 hours and, during the final 4 hours, incubated with 50 nM PMA and 0.5 μg/ml ionomycin (Sigma-Aldrich) along with GolgiStop and GolgiPlug (BD Biosciences). Following this, cell-surface staining for various molecules and chemokine receptors was conducted, followed by intracellular staining for different cytokines using the BD CytoFix/CytoPerm and Perm/Wash kit (BD Biosciences) according to the manufacturer’s instructions. APC-labelled antibodies to IL-17A (eBiosciences), IL-4 (eBiosciences), or CCR5 (BioLegend), as well as PE-labelled antibodies to IFNγ (BD Biosciences), IL-10 (BD Biosciences), or CXCR3 (BioLegend), were employed. Cells were analyzed using a FACSCanto II flow cytometer (BD Bioscience). The data were analyzed using FlowJo software (Tree Star).

### 2.9 Analysis of prostate infiltrating leukocytes

Analysis of prostate infiltrating leukocytes was conducted following previously established protocols (27, 29). In brief, the prostates were mechanically disrupted and enzymatically digested in RPMI 1640 medium containing 1 mg/ml collagenase D (Roche, Basilea, Switzerland) and 50 U/ml DNase I (Sigma-Aldrich) for 45 minutes at 37°C. Following digestion, the suspensions were filtered through 75- and 40-μm cell strainers (BD Biosciences), and single-cell suspensions were washed twice in RPMI 1640 medium supplemented with 10% FBS, 2 mM EDTA, and 50 mM 2-ME. Cells were then stained with various antibodies for flow cytometry analysis and acquired using a FACSCanto II. The data were analyzed using FlowJo software (Tree Star).

### 2.10 Assessment of chronic pelvic pain

The development of chronic pelvic pain was analyzed by behavioral testing as described previously (29, 46, 47). Mice were analyzed before immunization (baseline, day 0), and at 7, 14, and 23 days after the initial immunization. Tests were conducted in individual Plexiglas chambers featuring a stainless-steel wire grid floor, following an acclimation period. Referred hyperalgesia and tactile allodynia were evaluated using von Frey filaments with forces ranging from 0.04 to 4 g (Bioseb, Chaville, France). Each filament was applied for 1–2 s with an inter-stimulus interval of 5 s, totaling 10 times, and the filaments were applied in ascending order of force. Stimulation was confined to the lower abdominal area proximal to the prostate, and efforts were made to stimulate various areas within this region to prevent desensitization or “wind-up” effects. An investigator blinded to the treatment groups graded animal responses. Positive responses to filament stimulation were categorized as: 1) sharp retraction of the abdomen; 2) immediate licking or scratching of the stimulated area; or 3) jumping. Response frequency was calculated as the percentage of positive responses, and data were presented as the mean percentage of response frequency ± SEM.

### 2.11 Statistical analyses

Statistical analyses were conducted using one-way ANOVA with Bonferroni *post hoc* test analysis. Tactile allodynia testing results were analyzed using two-way ANOVA with Šídák’s multiple comparisons test *post hoc* analysis. Mean values along with their standard error of the mean (SEM) are depicted in the graphs. Statistical computations were carried out using GraphPad Prism 5.0 software. A significance level of *p < 0.05 was applied to all analyses.

## 3 Results

### 3.1 CD8 T cell deficiency does not impair the induction of mixed Th1/Th17 immune responses upon PA-immunization in EAP

**Figure 1:**
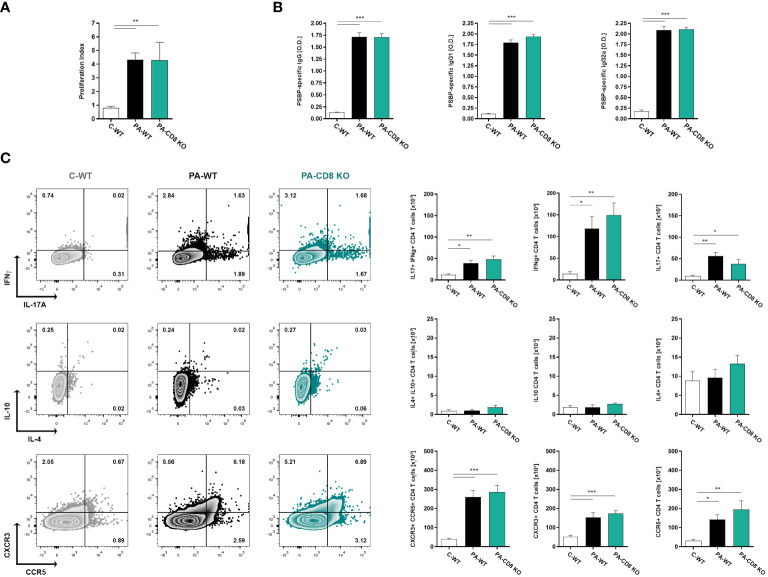
Mixed prostate-specific Th1/Th17 immune underlying EAP development are similarly induced upon PA-immunization in either CD8 KO or wild type mice. CD8 KO or C57BL/6 mice were immunized with PA emulsified in CFA (PA-CD8 KO and PA-WT groups) or with saline-CFA (control animals, C-WT). Mice were euthanized at day 24 after immunization, and sera and prostate draining lymph nodes were obtained. Prostate draining lymph node cells were cultured for 48 or 72 h in the presence of PSBP or medium. **(A)** PSBP specific lymphoproliferative responses from prostate-draining lymph node cells were expressed as proliferation index. **(B)** Serum levels of PSBP-specific total IgG, IgG1, and IgG2a, assessed by indirect ELISA (serum specimens diluted 1/200). **(C)** Representative flow cytometry dot plots of intracellular staining for IFNγ, IL-17A, IL-10 and IL-4, and of surface staining for CXCR3 versus CCR5, performed on prostate draining lymph node cells stimulated with PSBP for 48 h. Numbers indicate percentage among gated CD4+ T cells. Bar graphs depict absolute numbers of the indicated cytokine- or chemokine receptor-expressing CD4+ T cells. Data are shown as mean ± SEM, n=4–6 per group, and are representative of three independent experiments. Statistical analysis was performed using one-way ANOVA with Bonferroni post hoc test analysis. *p< 0.05, **p< 0.01, and ***p< 0.001.

With the aim of assessing whether the absence of CD8 T cells had an effect on the specific Th1/Th17 immune response underlying EAP pathogenesis and chronic pelvic pain development, we analyzed the induced immune response upon PA immunization in CD8α-deficient (CD8 KO) mice and in wild type (WT) mice. Animals were immunized and, 24 days after, the cellular and humoral specific immune responses specific to Prostatein or PSBP, the major target autoantigen in EAP (28, 41), were analyzed. As shown in **Figure 1**, comparable positive lymphoproliferative responses to PSBP were detected from prostate-draining lymph node cells from both groups of PA-immunized animals, either wild type (PA-WT) or CD8 KO (PA-CD8 KO) mice, when compared with control animals (C-WT, **Figure 1A**). Besides, these cellular immune responses were accompanied by comparable high serum levels of PBSP-specific total IgG, IgG1 and IgG2a in either PA-WT or PA-CD8-KO mice respect to control animals (C-WT, **Figure 1B**). To confirm whether the typical mixed prostate-specific Th1/Th17 immune responses underlying EAP induction were observed in our setting, intracellular cytokine staining was conducted on mononuclear cells from prostate-draining lymph nodes after 48 hours of *in vitro* stimulation with PSBP. Interestingly, the induction of similarly higher frequencies of IFNγ+ or IL-17A+ CD4 T cells was observed in both groups of PA-immunized mice (PA-WT or PA-CD8 KO) compared with control animals (C-WT, **Figure 1C**). Conversely, there were no significant differences in the frequencies of IL-10+ and/or IL-4+ CD4 T cells among experimental groups (C-WT, PA-WT and PA-CD8 KO, **Figure 1C**). Interestingly, significantly higher frequencies of autoreactive CD4 T cells expressing the chemokine receptors CXCR3 and CCR5 were detected in either PA-WT or PA-CD8 KO mice than in control animals (C-WT, **Figure 1C**).

These findings demonstrate that mice lacking CD8 T cells can generate comparable prostate-specific Th1 and Th17 immune responses to those observed in their wild-type counterparts during EAP induction.

### 3.2 The development of prostate chronic inflammation in EAP does not depend on CD8 T cells

**Figure 2:**
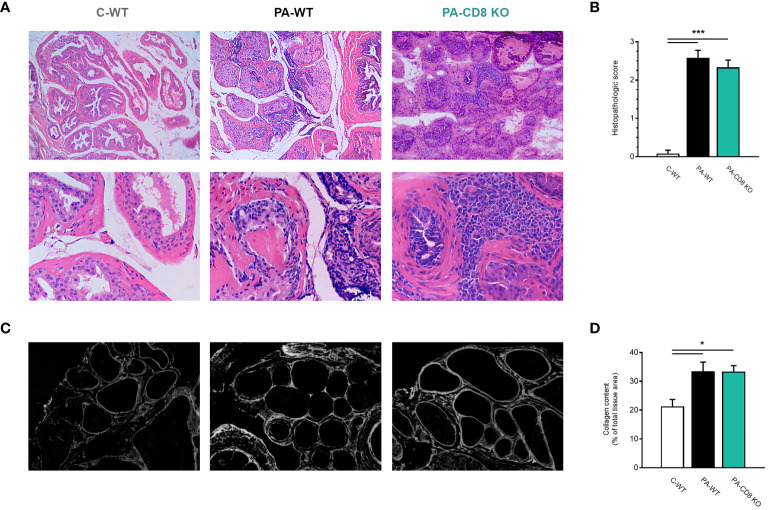
Mice deficient in CD8 T cells develop prostate chronic inflammation after EAP induction. CD8 KO or C57BL/6 mice were immunized with PA emulsified in CFA (PA-CD8 KO and PA-WT groups) or saline-CFA (control animals, C-WT). Mice were euthanized at day 24 after immunization and the prostate glands were excised and processed for histopathology analysis. **(A)** Representative hematoxylin and eosin stained histological assays in prostate tissue sections (original magnifications 100X and 400X), and **(B)** Prostate histopathological scores and from mice under study. **(C)** Representative picrosirius red (PSR) stained histological assays in prostate tissue sections, conducted to assess prostate collagen content (original magnification 400X). **(D)** Bar graph depicting collagen content quantification in prostate tissue from mice from the different experimental groups. Data are shown as mean ± SEM, n=4–6 per group, and are representative of three independent experiments. Statistical analysis was performed using one-way ANOVA with Bonferroni *post hoc* test analysis. *p< 0.05, and ***p< 0.001.

**Figure 3:**
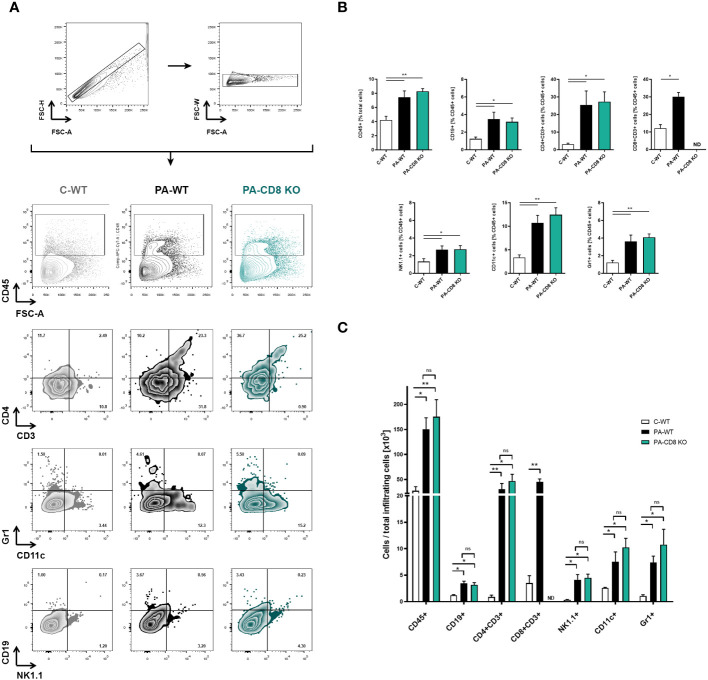
Prostate leukocyte infiltration is comparably induced in either CD8 KO or wild type after EAP induction. CD8 KO or C57BL/6 mice were immunized with PA emulsified in CFA (PA-CD8 KO and PA-WT groups) or saline-CFA (control animals, C-WT), and mice were euthanized at day 24 after immunization. The prostates were excised and processed for flow cytometry analysis. Live cells were counted and flow cytometry was preformed to evaluate leukocyte infiltration in the prostate glands from animals under study. **(A)** Gating strategy and representative flow cytometry density plots of the analysis of different leukocyte subpopulations: total leukocytes were gated on plots showing CD45+ cells versus forward scatter, and the different leukocyte cell subsets were analyzed in plots showing CD4+ versus CD3+ cells, Gr1+ versus CD11c+ cells, and CD19+ versus NK1.1+ cells. Bar graph depicts relative **(B)** or absolute numbers **(C)** of CD45+, CD19+, CD4+CD3+, CD8+CD3+, NK1.1+, CD11c+, and Gr-1+ cells in the prostate gland from mice under study. Numbers indicate the percentage of cells in each quadrant. Data are shown as mean ± SEM, n=4–5 per group, and are representative of three independent experiments. Statistical analyses were performed using one-way ANOVA with Bonferroni *post hoc* test analysis. *p< 0.05, and **p< 0.01; ns, non-significant; ND, not detected.

Then, the prostates from animal groups under study underwent histopathological analysis, while collagen deposition was measured as an indicator of chronic inflammation. As shown in **Figure 2A**, both groups of PA-immunized mice, PA-WT and PA-CD8 KO, developed similarly severe tissue damage and inflammation revealed by marked multifocal perivascular and stromal infiltration of mononuclear cells, oedema, hemorrhage, epithelial cell desquamation, and severe tissue disorganization were noted, resulting in EAP histopathological scores of 2.33 ± 0.65 and 2.58 ± 0.67, respectively (**Figures 2A, B**). As expected, no significant tissue alterations were observed in prostate tissue sections from control mice, yielding an EAP histopathological score of 0.08 ± 0.29 (**Figures 2A, B**). Moreover, staining with PSR, a collagen stain capable of binding to both fibrillar and non-fibrillar collagen subtypes (45), revealed significantly increased collagen deposition in prostate tissue sections from both groups of PA-immunized mice, PA-WT and PA-CD8 KO with respect to control animals (C-WT, **Figures 2C, D**). The latter suggested the presence of fibrosis, a fundamental element of chronic inflammation. In parallel, prostate infiltrating leukocytes were quantified and characterized by flow cytometry (**Figure 3**). In agreement with these findings, significantly higher amounts of total leukocytes (CD45+ cells) were observed in prostate tissue samples from both groups of PA-immunized mice, PA-WT and PA-CD8 KO, compared with control animals (approximately 3–4 fold increase), which exhibited minimal to no prostate leukocyte infiltration (C-WT, **Figures 3A-C**). The leukocyte infiltrates predominantly consisted of CD4+ T lymphocytes, Gr1+ granulocytes, and CD11c+ cells, while CD19+ B lymphocytes and NK cells were present in either lower proportions (**Figures 3B, C**). As expected, no prostate infiltrating CD8 T cells were detected in PA-CD8 KO mice, whereas significantly higher counts were observed in PA-WT mice than in control animals (C-WT, **Figures 3B, C**). These findings show that both, CD8 KO mice and wild type (C57BL/6) mice, exhibit similar types and extents of prostate tissue lesions and chronic inflammation upon EAP induction, indicating that CD8 T cells do not significantly contribute to EAP pathogenesis.

### 3.3 CD8 T cells are dispensable for chronic pelvic pain development

**Figure 4:**
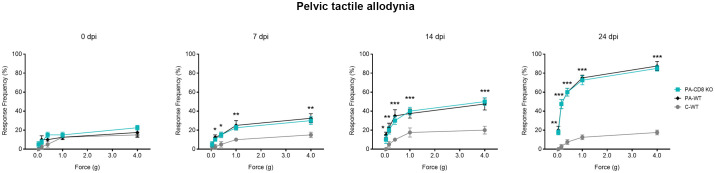
Chronic pelvic pain is developed by animals able to mount prostate-specific Th1 immune responses, even in the absence of CD8 T cells. CD8 KO or C57BL/6 mice were immunized with PA emulsified in CFA (PA-CD8 KO and PA-WT groups) or CFA alone (control animals, C-WT), and referred visceral hyperalgesia was measured over time of EAP induction as responses to mechanical stimulation of the pelvic region and hind paw using von Frey filaments of 5 calibrated forces. Data are shown as the mean percentage of response frequency ± SEM (e.g., 5 responses of 10 = 50%) before (baseline, Day 0) or at 7, 14, and 24 days after PA immunization. Tactile allodynia responses to pelvic stimulation of every experimental group under study over different time points of EAP induction. Shown data (n=4–5 per group) are representative of three independent experiments. Statistical analysis was performed using two-way ANOVA with Šídák’s multiple comparisons post hoc test analysis. *p< 0.05, **p< 0.01, and ***p< 0.001.

To evaluate whether the onset of prostate inflammation and leukocyte infiltration was accompanied by pelvic pain development, PA-CD8 KO and PA-WT mice along with control (C-WT) mice underwent suprapubic allodynia assessments at baseline and following PA immunization. Pelvic area mechanical stimulation in PA-immunized mice resulted in positive response frequencies correlating with the applied force (48), with similar response profiles observed in PA-CD8 KO and PA-WT mice (**Figure 4**). Significant increases in tactile allodynia were noted at both early (7 dpi) and late (14 and 24 dpi) time points in either PA-CD8 KO or PA-WT mice, indicating comparable chronic pelvic pain development kinetics (**Figure 4**). Altogether, these findings indicate that CD8 T cells do not play a pivotal role in the induction and progression of prostate inflammation and the development of chronic pelvic pain in EAP.

## 4 Discussion

CPPS/CP is an urological condition that poses a significant healthcare challenge due to its considerable incidence among young and middle-aged men and it significant deleterious impact in the quality of life of patients (2–6, 11, 49–51). Furthermore, its aetiology remains largely unknown, leading to empirically prescribed therapies that often yield suboptimal clinical outcomes, exacerbating patient discomfort and dissatisfaction (1, 5, 9–11, 50, 52, 53). Accumulating evidence suggests that the syndrome is likely a result of dysregulated inflammation manifested through autoimmunity targeting more than one prostate autoantigen, a typical feature of autoimmune diseases. Indeed, several studies have reported evidence indicating an autoimmune aetiology for CPPS/CP, as a significant proportion of patients display self-reactive T-cell responses, notably Th1 and Th17, targeting various prostate antigens coupled with Treg dysfunction. These responses correlate with inflammation in semen and the prostate, in the absence of detectable infections (8, 10, 12, 19–24, 54–59). Furthermore, treatment with immunosuppressants significantly improves CPPS/CP symptoms (53, 60). Moreover, rodent models of EAP have provided compelling evidence supporting the autoimmune basis of CPPS/CP (17, 55, 61, 62). EAP models have been extensively used to investigate the pathogenesis of CPPS/CP, demonstrating reliability and validity as they closely mirror key findings observed in patients, having provided fundamental evidence about the immune mechanisms underlying disease and chronic pelvic pain development and the associated pathological consequences (10, 14, 17, 61, 62). After immunization with a mixture of PA, purified prostate proteins such as PSBP, or peptides, animals develop mixed PA-specific T cell and antibody responses that associate with florid histologic prostatitis, and the development of chronic pelvic pain (17, 61, 62). As in patients, mice strains susceptible to EAP typically develop mixed PA-specific Th1 and Th17 immune responses, semen and/or prostate inflammation, and chronic pelvic pain (25, 26, 41, 42, 46, 63). Interestingly, a fundamental role for Th1 cells in driving disease pathogenesis and chronic pelvic pain development was already shown (27, 29, 64). In fact, the adoptive transfer of sorted PA-specific CD4 Th1 lymphocytes expressing the associated chemokine receptors CXCR3 and CCR5 was sufficient to induce histological prostatitis, with the local expression of several cytokines and chemokines, which subsequently recruited more leukocytes worsening prostate inflammation and inducing chronic pelvic pain (27). Prostate tissue inflammation in EAP is characterized by significant infiltration of macrophages, helper and cytotoxic T cells, granulocytes, and mast cells, and the expression of inflammatory cytokines and chemokines such as IFNγ, IL-17, IL-12, TNF, IL-1β, CXCL9, CXCL10, CXCL11, CCL2, CCL3, CCL4, and CCL5 (26, 27, 41, 42, 64). Interestingly, mast cells, their chemoattractants CCL2 and CCL3, and the tryptase-PAR2 axis have shown to be essential for chronic pelvic pain development and maintenance (42, 65–67). Once again, these findings mirror the human disease, since elevated levels of CCL2 and CCL3, the Th1-associated chemokine CXCL10, mast cell-derived tryptase and other mediators, and significantly increased effector Th1/Th17 cell proportions have been found elevated in expressed prostatic secretions or peripheral blood from CPPS/CP patients, and correlating with pelvic pain symptoms (32, 66–71). Remarkably, recently reported data indicates that a mast cell-directed therapy improved the quality of life of CPPS/CP patients by alleviating pain and urinary symptoms in association with a downregulation of immune-related pathways including Th1 and Th17 T cell differentiation (72). From all these evidence, IFNγ-secreting Th1 lymphocytes emerge as central drivers in the pathogenesis of the disease and the development of chronic pelvic pain, the characteristic symptom observed in CPPS/CP patients. Nonetheless, the potential contribution of other fundamental components of the adaptive immune response, such as CD8 T cells, to disease development and pelvic pain induction and/or amplification remains largely unexplored. In that regard, Ye et al. reported significantly decreased levels of CD8 T cells in peripheral blood from CPPS/CP patients than in healthy control individuals (73). On the contrary, an elevation of CD8 T cells in semen from CPPS/CP patients has been described (24). Although the significance of these findings remains uncertain, one possible explanation is that CD8 T cells may be recruited to the prostate during an ongoing local inflammatory process, subsequently leading to an increase in their presence in semen. Interestingly, recently reported data by Zhang et al. suggest that while there were no significant differences in the proportions of peripheral blood naïve, central memory, effector memory, or terminally differentiated effector cells (TEMRA) CD8 T cell subsets between CPPS/CP patients and healthy controls, the CD8 TEMRA cell subset from CPPS/CP patients exhibited significantly elevated expression of IFNγ and TNF and an enriched inflammatory pathway signature compared to controls (74). Although very interesting, these results remain to be confirmed by further research since only 2 CPPS/CP patients and 2 healthy controls were analyzed (74). Therefore, the role of CD8 T cells in the pathogenesis of CPPS/CP remains uncertain and warrants further investigation.

An increasing body of evidence indicates that CD8 T cells play pivotal roles in the initiation, progression, pathogenesis, and protection for autoimmune diseases (75, 76). In that regard, it is noteworthy to highlight that CD8 T cells can exert not only direct cytotoxic effects but also a myriad of actions, including indirect cytotoxicity, as well as direct and indirect non-cytotoxic functions via crosstalk with other immune and non-immune cells (35). These actions include the recruitment of other immune cells to inflammation sites, the activation of NK cells and dendritic cells to ensure antigen presentation and the induction of effector innate and adaptive immunity (mainly in an IFNγ dependent manner), the help to B cells to support antibody production, immunoregulation, and the induction of tissue healing and regeneration (35, 76, 77). Interestingly, memory CD8 T cells were shown to protect dendritic cells from the killing by effector cytotoxic T cells via the upregulation of an endogenous inhibitor of granzymes, thus enhancing antigen presentation and thereby augmenting the consequent induction of Th1 immune responses (78). Remarkably, effector memory CD8 T cells express T-bet and secrete Th1-associated cytokines such as IFNγ, and chemokines including CCL3, CCL4 and CCL5, and express the chemokine receptor CXCR3 (75, 79). Given the significant implications of these mediators in CPPS/CP and chronic pelvic pain, and the documented elevation of CD8 T cells in semen from CPPS/CP patients and in the prostates of animals with EAP (24, 29, 31, 41, 42), different possibilities arise. On the one hand, CD8 T cells may play a central role in EAP pathogenesis and chronic pelvic pain development. Alternatively, rather than pathogenic, their recruitment to and infiltration of the prostate (and consequently their increase in semen) could represent an amplification of a Th1-driven ongoing inflammatory process in the prostate, where elevated levels of cytokines and chemokines that bind to their receptors (e.g. CXCR3) are secreted. Finally, it is also possible that both scenarios are occurring simultaneously. To assess these possibilities, in our study we analyzed the induction of EAP and the development of chronic pelvic pain in CD8 KO and wild type mice. As already reported (26, 27, 29), our findings revealed that, following PA immunization, wild-type mice elicited prostate-specific Th1 and Th17 immune responses, leading to chronic inflammation of the prostate, characterized by hemorrhage, epithelial cell desquamation, marked periglandular leukocyte infiltration, and increased collagen deposition. Furthermore, these animals exhibited markedly heightened tactile allodynia responses as the disease advanced, indicating that the development of chronic pelvic pain was a result of inflammation of the prostate (26, 27, 29). Interestingly, comparable results were obtained when analyzing immunized CD8 KO mice. In fact, similarly elevated PA-specific Th1 and Th17 immune responses were detected in PA-immunized CD8 KO animals. Moreover, even in the absence of CD8 T cells, immunized animals developed significant chronic inflammation of the prostate and pelvic pain. Therefore, these results indicate that CD8 T cells do not play a major role in EAP pathogenesis and chronic pelvic pain development. Our findings are in line with the limited reported data regarding the involvement of CD8 T cells in prostate inflammation and the development of chronic pelvic pain. Our data support previously reported findings indicating that NOD mice lacking expression of beta 2-microglobulin are as susceptible to EAP as their wild type counterparts, indirectly indicating that CD8 T cells are dispensable for EAP pathogenesis (28). Moreover, our results corroborate previous data showing that, after PA immunization, a CD4 Th1 associated immune response develops and drives the induction of prostate tissue inflammation and chronic pelvic pain (10, 12, 29, 70). Collectively, these findings support the notion that CD8 T cells are not pivotal for EAP and chronic pelvic pain development. Instead of being inherently pathogenic, the observed increase in CD8 T cells in the semen of CPPS/CP patients and in the prostates of animals with EAP might reflect an amplification of the established Th1-driven inflammatory process in the prostate during EAP. Given that effector memory CD8 T cells express the chemokine receptor CXCR3 (75, 79), and their cognate ligands are elevated in the prostate of mice with EAP and in semen from patients with CPPS/CP (24, 29, 31, 41, 42), CD8 T cells would be recruited as a consequence or the ongoing inflammatory process in the prostate rather than being pathogenic. Remarkably, there are no reported studies assessing the direct role of CD8 T cells in the pathogenesis of EAP and the development of chronic pelvic pain. Thus, we consider the present work provides novel evidence that add to the current knowledge in the field. However, future research employing other complementary experimental approaches beyond the use of CD8α KO and wild-type C57BL/6 mice, and assessing their putative link with cells involved in pelvic pain development such as mast cells, may be necessary to confirm our findings.

In summary, we provided compelling data showing that CD8 T cells do not play a major role in EAP pathogenesis and chronic pelvic pain development. Moreover, our results corroborate the previous notion that, after PA immunization, a CD4 Th1 associated immune response develops and drives the induction of prostate tissue inflammation and chronic pelvic pain. These findings constitute a significant contribution to the field and improve the current knowledge about the immune mechanisms underlying EAP, a reliable experimental model for the study of CPPS/CP, a prevalent clinical condition in which the precise aetiology and pathogenesis remain to be unveiled.

## Data availability statement

The original contributions presented in the study are included in the article/supplementary material. Further inquiries can be directed to the corresponding author.

## Ethics statement

The animal study was approved by Institutional Laboratory Animal Care and Use Committee (Comité Institucional para el Cuidado y Uso de Animales de Laboratorio - CICUAL) at Facultad de Ciencias Químicas, Universidad Nacional de Córdoba, Cordoba, Argentina. The study was conducted in accordance with the local legislation and institutional requirements.

## Author contributions

FS: Formal Analysis, Investigation, Methodology, Validation, Visualization, Writing – review & editing. MM: Data curation, Formal Analysis, Investigation, Methodology, Software, Validation, Writing – review & editing. DP: Data curation, Formal Analysis, Investigation, Methodology, Software, Validation, Visualization, Writing – review & editing. YC: Data curation, Formal Analysis, Investigation, Methodology, Software, Validation, Visualization, Writing – review & editing. CO: Data curation, Formal Analysis, Investigation, Methodology, Software, Validation, Visualization, Writing – review & editing. GG: Data curation, Formal Analysis, Investigation, Methodology, Software, Validation, Visualization, Writing – review & editing. EA-R: Funding acquisition, Resources, Writing – review & editing. VR: Conceptualization, Funding acquisition, Resources, Supervision, Writing – review & editing. RM: Conceptualization, Formal Analysis, Funding acquisition, Investigation, Project administration, Resources, Software, Supervision, Validation, Visualization, Writing – original draft, Writing – review & editing.
